# Quality of life after cardiac surgery in an octogenarian population

**DOI:** 10.1186/cc9432

**Published:** 2011-03-11

**Authors:** M Nydegger, A Boltres, K Graves, A Zollinger, CK Hofer

**Affiliations:** 1 Triemli City Hospital, Zurich, Switzerland

## Introduction

An increasing number of cardiac surgery procedures are performed today in patients >80 years [1]. However, only limited data are available regarding the postoperative outcome in this patient group. The aim of this study was to assess quality of life in patients >80 years after elective cardiac surgery (CS80) compared with younger patients (60 to 70 years; CS60).

## Methods

Consecutive CS80/CS60 patients during a 1-year period were contacted 12 months after cardiac surgery. A structured interview was performed and quality of life was assessed (SF-36 health survey). Norm-based scoring (transformed to mean = 50 ± 10) was analysed. Sociodemographic and procedure-related data were obtained from the hospital database. Student's *t*-test and the chi-square test were used to compare both groups.

## Results

**Figure 1:**
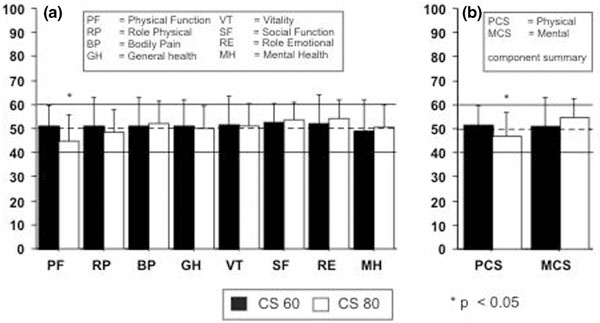
**Norm-based SF-36 scoring profile: **(a) **single components and **(b) **component summaries**.

Fifty-three and 52 datasets for CS80 and CS60, respectively, were available for statistical analysis: mean age was 82.2 ± 2.7 years (CS80) and 64.7 ± 2.7 years (CS60, *P *< 0.001). There was no significant difference of preoperative cardiac function or risk score (ejection fraction: CS80: 54 ± 14%, CS60: 54 ± 13%; *P *= 0.78. Euroscore: CS80: 9.3 ± 0.24, CS60: 6.9 ± 3.7, *P *= 0.09). ICU length of stay was 5.3 ± 9.1 days (CS80) and 2.6 ± 2.7 days (CS60, *P *< 0.04); hospital length of stay was 15.6 ± 10.1 days (CS80) and 15.1 ± 8.5 days (CS60, *P *= 0.79). The 30-day mortality rate was 11.5% (CS80) and 5.6% (CS60, *P *= 0.27), and 1-year mortality was 16.3% (CS80) and 7.6% (CS60, *P *= 0.13). SF-36 physical and mental health components ranged from 44.8 ± 10.8 to 54.2 ± 7.6 (CS80) and from 48.7 ± 13.5 to 52.7 ± 7.9 (CS60; Figure 1); physical function (PF) was significantly lower for CS80 (*P *= 0.002). Physical component summary (PCS) was 46.9 ± 9.9 (CS80) and 51.3 ± 8.8 CS60; *P *= 0.03); mental component summary (MCS) was 54.7 ± 7.9 (CS80) and 50.8 ± 12.0 (CS60; *P *= 0.75; Figure 1).

## Conclusions

Quality of physical health with only minor limitations was observed in patients after cardiac surgery aged >80 years as compared with younger patients (60 to 70 years). There was no difference of mental health quality between both patient groups. These results could only be achieved with increased ICU length of stay for patients >80 years.
